# The Antibacterial Effect and Mechanism of a Bacteriocin Produced by *Weissella sagaensis* ZD-66 against Major Foodborne Pathogens

**DOI:** 10.4014/jmb.2507.07006

**Published:** 2025-09-16

**Authors:** Sijia Sun, Xinling Zhao, Changjiang Wang, Guo Pan, Lirong Yang, Wanli Sha, Wenlong Dong, Baishuang Yin

**Affiliations:** 1 College of Animal Science and Technology, Jilin Agricultural Science and Technology University, Jilin 132000, P.R. China; 2 Jilin Provincial Key Laboratory of Preventive Veterinary Medicine, Jilin 132000, P.R. China; 3 Jilin Province Technology Innovation Center of Pig Ecological Breeding and Disease Prevention and Control, Jilin 132000, P.R. China; 4 Jilin Province Cross Regional Cooperation Technology Innovation Center of Porcine Main Disease Prevention and Control, Jilin 132000, P.R. China

**Keywords:** Bacteriocin, *Weissella sagaensis*, *Salmonella* Typhimurium, antibacterial mechanism

## Abstract

*Salmonella* Typhimurium is a pathogen bacterium responsible for foodborne diseases. In this study, *Weissella sagaensis* ZD-66, a bacteriocin-producing strain, was isolated from pickled vegetables. The bacteriocin produced by this strain was characterized, and its antibacterial mechanism was investigated. The genome size of *W. sagaensis* ZD-66 was 1,862,304 bp, with a GC content of 36.87%. Further, bioinformatic analysis using the BAGEL4 database revealed a class IIa bacteriocin gene cluster, identified as Penocin A. *W. sagaensis* ZD-66 showed maximum antibacterial activity at 28 h during fermentation. The *W. sagaensis* ZD-66 was partially purified by using dialysis and Tricine-SDS-PAGE, which revealed a molecular weight of less than 1 kDa. The *W. sagaensis* ZD-66 showed broad-spectrum inhibition against foodborne pathogens, including *S*. Typhimurium, *Staphylococcus aureus*, *Escherichia coli*, *Listeria monocytogenes*, and *Cronobacter sakazakii*. The bacteriocin was stable at 121°C for 30 min, pH resistant (pH 3-7), and under UV exposure for up to 2.5 h. However, its antibacterial activity of bacteriocin decreases after treatment with pepsin, trypsin, and pronase E. Furthermore, the treatment with bacteriocin led to the cell membrane being damaged, nucleic acid leakage, and reduced intracellular ATP content. Observation of the ultrastructure of bacteria by Scanning electron microscopy revealed that bacteriocin can inhibit *S*. Typhimurium by disrupting the cell membrane and pore formation. These results suggest that the bacteriocin produced by *W. sagaensis* ZD-66 has the potential for a natural food preservative.

## Introduction

Food poisoning caused by contamination and foodborne pathogens is a serious problem [1]. *Salmonella* Typhimurium (*S*. Typhimurium) is a Gram-negative bacterium and one of four primary causes of diarrhoeal diseases [2]. *S*. Typhimurium is one of the causes of diarrhoeal diseases that has been discovered in milk, eggs, vegetables, water, raw meats, and poultry [3-5]. In 2019, the European Union (EU) reported 87,923 cases of salmonellosis, resulting in nearly 140 deaths [6]. Clinically, antibiotics are currently the primary treatment for salmonellosis [7]. However, the antibiotic resistance of *S*. Typhimurium continues to increase, which poses a substantial threat to public health [8].

With the increasing awareness of food safety, consumer demand to reduce the use of food preservatives and food additives [1]. Thus, there is a need for the development of natural antibacterial agents. Lactic acid bacteria (LAB) bacteriocins have attracted wide attention because they are considered to be potential food preservatives [9]. Bacteriocins are protein or antimicrobial peptides composed of ribosomes [10]. LAB bacteriocins are safe, heat resistant, and easily inactivated by protease [11]. The LAB bacteriocins are classified into three main classes. Class I bacteriocins are post-translationally modified and heat-stable small peptides (<5 kDa), containing characteristic polycyclic thioether amino acids lanthionine or methyllanthionine, as well as unsaturated dehydroalanine amino acids and 2-aminoisobutyric acid. Class II bacteriocins consist of non-modified small heat-resistant peptides (<10 kDa) with amphiphilic helical structure with a conserved N-terminal sequence YGNGV-C, and the representative bacteriocins include leucocin A, acidocin A, pediocin PA-1 and lactococcin G *et al*. Class III bacteriocins consists of heat-labile proteins with large molecular weight (>30 kDa) [12, 13]. Nisin is the most famous LAB bacteriocin, and it is permitted for use in food by the FDA and EU [14]. Although Nisin has been widely used as a natural food protectant, it still has some shortcomings, such as the influence of pH on its bacteriostatic activity [15]. Therefore, it is necessary to identify a broad-spectrum and stable bacteriocin.

Some bacteriocins have been reported as natural food preservatives in the food industry. For example, *Pediococcus pentosaceus* M46 inhibits the formation of *S*. Typhimurium biofilm [16]. *Lactobacillus plantarum* A6 and *Lactobacillus fermentum* Ogi E1 have been reported to strongly inhibit *Salmonella* strains [3]. The *Lactobacillus brevis* DF01 strain has been demonstrated to reduce the formation of *S*. Typhimurium biofilms by nearly 47% [17]. The minimum inhibitory concentration of *Lactobacillus paracasei* ZFM54 against *S*. Typhimurium is 3.50 ug/ml [18], which is considered a relatively low concentration level. Even though many antimicrobial peptides and proteins have been extensively studied, there is still a lack of relevant evidence for their application in the field of food [19]. People have conducted extensive research on LAB bacteriocins, but there is currently less research on *Weissella sagaensis* (*W. sagaensis*).

The aim of the present study was to extract a bacteriocin from *W. sagaensis* ZD-66, which has shown strong antibacterial activity against *S*. Typhimurium. In order to gain further insight into bacteriocin coding genes in *W. sagaensis* ZD-66, the genome was sequenced for this study. Further, we investigate the mechanism of bacteriocin.

## Materials and Methods

### Isolation and Screening of Bacteriocin Producing Strain

The marinated vegetables samples from Jilin Province (China) were serially diluted with sterile physiological water (0.85% NaCl) to 10^-2^, plated on MRS agar (Hopobio, China), and incubated for 24 h at 37°C. After purification by streaking, LAB strains were subjected to preliminary identification using Gram staining. Among these, ZD-66 was isolated from the pickled vegetables in Jilin Province (China), and inhibitory substances produced by ZD-66 showed the highest antibacterial activity against *S*. Typhimurium ATCC 14028. Therefore, ZD-66 was chosen as the object of further research, and the antibacterial substance produced by strain ZD-66 was named ZD-66B. The strains ZD-66 were frozen stocks at -80°C in 20% (v/v) glycerol.

### Preparation of Bacteriocin Sample and Detection of Antibacterial Activity

The strain ZD-66 was cultured in 300 ml of MRS broth (Hopobio) at 37°C for 48 h, after which the culture solution was centrifuged at 3,625 ×*g* for 15 min at 4°C using a TGL16M centrifuge (Yancheng Kate Experimental Instrument Co., Ltd., China). Thereafter, the supernatant was filtered through a 0.22 μm sterile filter to collect the cell-free supernatant (CFS). The obtained CFS was adjusted to pH 6.5 with 1 N NaOH to exclude the interference of organic acids [20]. Catalase was added to CFS to a final concentration of 1 mg/ml, and the samples were incubated at 37°C for 2 h. Then, the catalase was terminated by 100°C for 5 min [21]. Untreated CFS was used as a control. *S*. Typhimurium ATCC 14028 (1 × 10^6^ CFU/ml) was used as an indicator bacteria. The antimicrobial activity was determined by using the Oxford cup method as described by Li *et al*. [22]. An 8 mm sterilized Oxford cup was placed vertically on MH agar, 2 ml *S*. Typhimurium ATCC 14028 (1 × 10^6^ CFU/ml) was added to 100 ml of the MH agar, which was then poured onto the surface of the bottom MH agar. After solidification, pull out the Oxford cup and add 100 ul bacteriocin into it. The plates were placed incubation at 37°C for 12 h. Measured the diameter of the inhibition zone using a vernier calipers.

### 16S rRNA Gene Sequencing of ZD-66

The strain ZD-66 genomic DNA was extracted using a bacterial genomic DNA kit (Aidlab Biotechnologies Co. Ltd., China). Amplification of the 16S rRNA by polymerase chain reaction (PCR) using universal primers (27F: 5'-AGAGTTTGATCCTGGCTCAG-3' and 1492R: 5'-TACGGTTACCTTGTTACGACTT-3'). Sanger sequencing of PCR products was performed by Comate Bioscience Co. Ltd., (China). Homology searches on the obtained sequence were conducted using the NCBI (https://blast.ncbi.nlm.nih.gov/Blast.cgi). Phylogenetic analysis was reconstructed using MEGA11. The phylogenetic tree was visualized by using Chiplot (https://www.chiplot.online/).

### Whole Genome Sequencing of *W. sagaensis* ZD-66

The *W. sagaensis* ZD-66 was cultured in MRS broth at 37°C for 24 h, and the genomic DNA extracted was used for whole-genome sequencing. The filtered reads were assembled using Canu v1.5 software for subsequent genomic analysis. Coding genes were predicted using Prodigal v2.6.3. Repetitive sequences were predicted using RepeatMasker v4.0.5 and predicted ribosomal RNA (rRNA) using Infernal v1.1.3. Bacteriocin coding gene clusters were predicted using BAGEL4, and the potential types of bacteriocins produced by the bacterial strain were identified. The bacteriocin coding gene cluster was visualized by using IBS [23].

### Growth Curve of ZD-66

The growth curve of *W. sagaensis* ZD-66 was assessed based on the research of Leong *et al*. with slight modifications [24]. *W. sagaensis* ZD-66 was cultured in 2% (v/v) inoculation rate into MRS broth at 37°C for 30 h. Samples were taken every 2 h, and the absorbance at 600 nm were measured. The culture solution was centrifuged at 7,378 ×*g* for 5 min (Sigma, Germany) to gain CFS. Then, the collected CFS was assessed the antibacterial activity by the Oxford cup method using *S*. Typhimurium ATCC 14028 as indicator bacteria.

### Partial Purification of Bacteriocin and Estimation of Molecular Mass

*W. sagaensis* ZD-66 was cultured in 2% (v/v) inoculation rate into 1,200 ml MRS broth at 37°C for 28 h, and was obtained CFS using the method described in section 2.2 above. The collected CFS was concentrated 10-fold at 45°C using a rotary evaporator. Samples were dialyzed using a 1,000 Da dialysis membrane (Beyotime, China) at 4°C for 24 h, and the liquid obtained after dialysis was concentrated 10-fold, and assayed for antibacterial activity analysis [22]. The molecular size of the partial purification bacteriocin was subjected to tricine-SDS-PAGE. The stacking gel comprised a 4% acrylamide, the spacer gel of 10% acrylamide, and the separating gel of 18% acrylamide. The dialyzed samples were mixed with sample buffer and boiled at 100°C for 10 min. The electrophoresis was initially performed at 30 V for 10 min, followed by an increase to 80 V until the interlayer gel was fully formed. Subsequently, the voltage was further increased to 120 V to ensure the tracer dye migrated to the bottom of the gel. After electrophoresis, one part of the gel was fixed in a fixing solution (50% methanol, 10% acetic acid and 40%distilled water) for 30 min, stained with Coomassie Brilliant Blue R-250 and destained in a de-staining solution (20% ethanol absolute, 10% glacial acetic acid and 70% distilled water) until clear protein bands were visible. The other part of the gel was washed three times with distilled water and then overlaid with MH agar inoculated with *S*. Typhimurium ATCC 14028 (1 × 10^6^ CFU/ml). The plate was incubated for 24 h at 37°C and then examined for an inhibition zone.

### Antimicrobial Spectrum of ZD-66B

The antimicrobial activity of crude purified bacteriocin ZD-66B was assessed against multiple foodborne pathogens using the Oxford cup method as described in section 2.2. Indicators included three Gram-negative bacteria (*Escherichia coli* ATCC 25922, *S*. Typhimurium ATCC 14028, *Cronobacter sakazakii* SSJ-127) and two Gram-positive bacteria (*Staphylococcus aureus* ATCC 25923, *Listeria monocytogenes* ATCC 19115). *E. coli* ATCC 25922, *S*. Typhimurium ATCC 14028, *C. sakazakii* SSJ-127, and *S. aureus* ATCC 25923 were cultured in LB broth (Hopobio) for 12 h at 37°C, while *L. monocytogenes* ATCC 19115 was cultured in BHI broth (Hopobio) for 12 h at 37°C. The indicator bacteria were subsequently diluted to a concentration of 1 × 10^6^ CFU/ml. Measured the diameter of the inhibition zone using a vernier calipers.

### Physico-Chemical Characteristics of ZD-66B

To evaluate the pH stability, the pH was adjusted to 3, 5, 7, 9, 11 with 1 N NaOH and HCl. After 1 h incubation at 37°C, the pH was adjusted back to the original value. To evaluate the effects of proteolytic enzymes, the crude purified bacteriocin ZD-66B was incubated at 37°C for 2 h with proteinase K, pronase E, pepsin, papain, and trypsin, with all enzymes adjusted to a final concentration of 1 mg/ml. Then, inactivate these enzymes for 5 min heated at 100°C. The ZD-66B was heated at 40°C, 60°C, 80°C, 100°C for 1 h and 121°C for 30 min to assess its thermo-stability. To evaluate the stability under UV light, ZD-66B was exposed to UV light for 0.5, 1.0, 1.5, 2.0 and 2.5 h [19, 21, 25]. The ZD-66B were unexposed to pH, enzymes, temperatures, and UV rays and were used as the control group. The antimicrobial activity was determined using the Oxford cup method.

### Mode of Action of ZD-66B

**The time-kill curves.** The time-kill curves were evaluated according to Yan *et al*. [26]. Briefly, *S*. Typhimurium ATCC 14028 was cultured to the exponential phase (1 × 10^8^ CFU/ml) at 37°C, and 10 ml of partially purified bacteriocin ZD-66B was mixed with 10 ml bacterial cultures for 24 h. Samples were taken every 2 h, and the absorbance at 600 nm was measured. PBS (Servicebio, China; 0.01 M, pH 7.5) was used as a negative control under identical treatment conditions.

**Measurement of the intracellular ATP content.** Measurement of the intracellular ATP in *S*. Typhimurium ATCC 14028 to assess of cytoplasmic membrane permeability, was measured as described by Rao at *et al*. [27]. The *S*. Typhimurium ATCC 14028 (1 × 10^8^ CFU/ml) was incubated with an equal volume of partially purified bacteriocin ZD-66B at 37°C for 0.5, 1.0, and 2.0 h, the culture was then centrifuged at 8,000 ×*g* for 15 min at 4°C, and the supernatant was discarded. PBS was used as a control in the same way treatment. The obtained bacterial sediment was added with 1 ml of extract, and subjected to ultrasonic fragmentation in an ice water. Ultrasonic disruption was performed using an ultrasonic cell crusher (Ningbo Science Biotechnology Co. Ltd., SCIENTZ-IID, China) at 200 W, ultrasound for 2 s, stop for 1 s, total ultrasound for 1 min. Afterward, the solution was centrifuged at 10,000 ×*g* at 4°C for 3 min. The ATP content was detected using an ATP detection kit (Solarbio, China) according to the manufacturer’s instructions.

**Propidium iodide (PI) uptake.** To clarify the antibacterial mechanism of partially purified bacteriocin ZD-66B, PI uptake analysis was performed according to Qiao *et al*. [28]. The *S*. Typhimurium ATCC 14028 was cultured to the exponential phase (1 × 10^8^ CFU/ml), centrifuged at 7,378 ×*g* at 4°C for 5 min, and resuspended in LB broth (Hopobio). Subsequently, the ZD-66B was added to the bacterial suspension at 37°C for 0.5, 1.0, and 2.0 h, then suspended and washed with PBS. The same treatment with PBS served as controls. The obtained mixture was incubated with PI (2 mg/ml, Sangon) at 37°C for 20 min in the dark. The PBS buffer was treated using the same method as the control group. The sample was observed under an fluorescence microscope.

**Scanning electron microscopy (SEM).** To evaluate the influence of partially purified bacteriocin ZD-66B on ultrastructural changes of *S*. Typhimurium ATCC 14028, the method was described by Xue *et al*. [21]. *S*. Typhimurium ATCC 14028 (1 × 10^8^ CFU/ml) by centrifugation (7,378 ×*g* at 4°C for 5 min) was washed three times with PBS buffer. The ZD-66B was exposed to an equal volume of bacterial suspension for 2.0 h at 37°C, then the same method for centrifugation and rinsing. PBS treatment was used as a control. Subsequently, the cells were fixed with 2.5% glutaraldehyde (Sangon, China) overnight at 4°C, and dehydrated using a series of gradient ethanol solutions (50%, 70%, 90%, and 100%) at 4°C. The dried samples were coated with gold-palladium and visualized by SEM.

### Statistical Analysis

All experiments were performed in triplicate. The results were presented as mean values ± standard deviation (SD). Statistical analysis was performed using one-way ANOVA and Duncan’s test using IBM SPSS Statistics 27.0. *P*-values < 0.05 were considered significant.

## Results

### Isolation and Identification of Lactic Acid Bacteria Strains

**Fig. 1:**
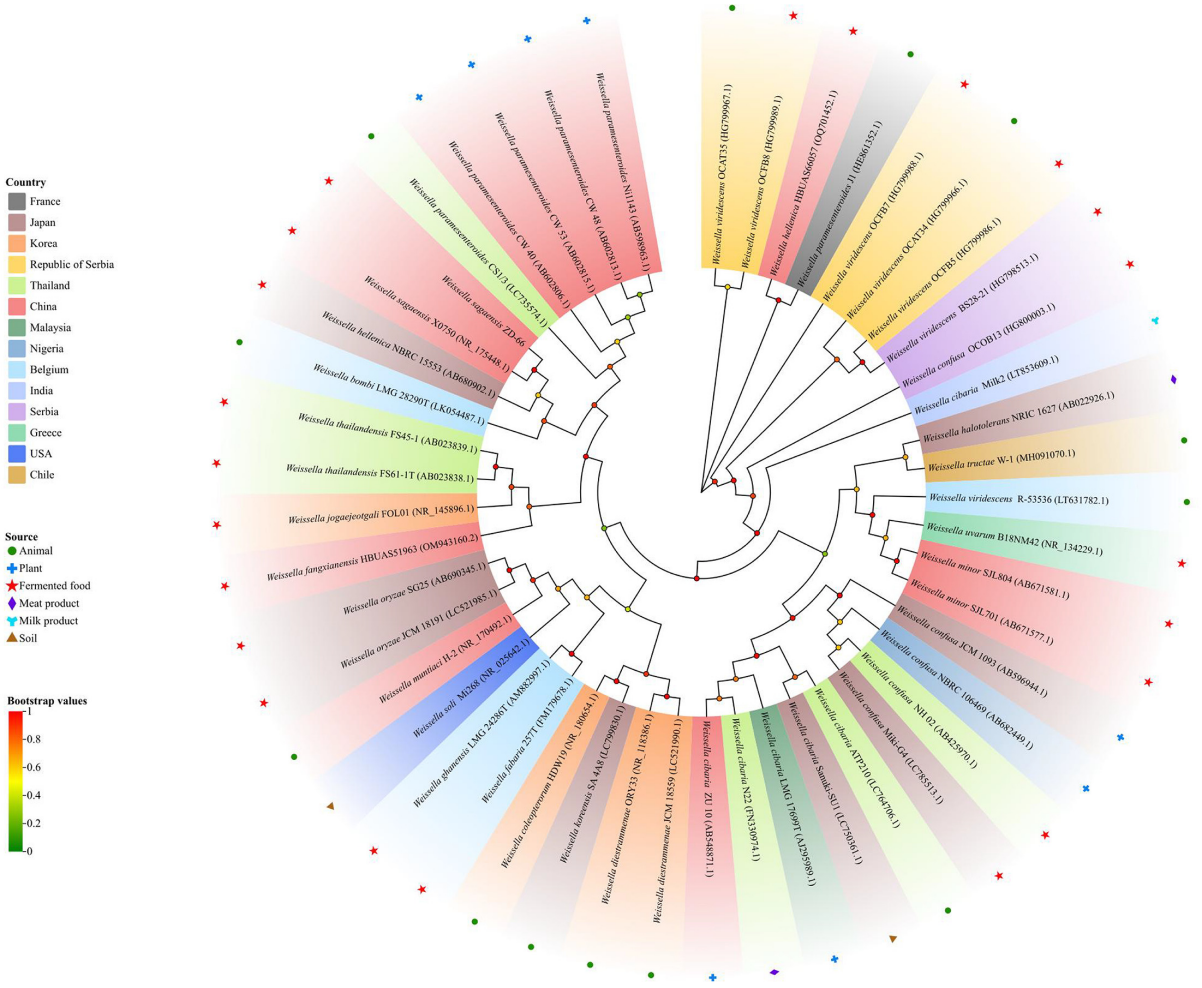
Phylogenetic tree based on 16S rRNA sequence of strain ZD-66. The phylogenetic tree was divided into fourteen groups, each color represents a different country. Six different graphics represent different sources.

The colony morphology of ZD-66 cultured on MRS agar plates at 37°C for 24 h exhibited a creamy white globular with a smooth surface. Microscopic analysis revealed that ZD-66 was a Gram-positive, short-rod bacterium. BLAST analysis of 16S rRNA gene sequence revealed that ZD-66 (GenBank: PQ867228) and *W. sagaensis* strain X0750 (GenBank: NR 175448.1) share 100% homology. This may be related to the fact that they all come from fermented food. The phylogenetic tree further proves a close cluster with ZD-66 strain and *W. sagaensis* strain X0750 (Fig. 1). Based on the above phenotypic and molecular studies, ZD-66 was identified as *W. sagaensis*.

### Evaluation of Antimicrobial Activity

The CFS adjusted to pH 6.5 had no antibacterial activity, and the catalase treatment did not decrease antimicrobial activity. These results indicated that the main inhibitory substances were neither organic acids nor hydrogen peroxide.

### Genome Properties and Bacteriocin Coding Gene Mining of *W. sagaensis* ZD-66

The raw sequencing data of ZD-66 were deposited in the NCBI database under the accession number CP166295. The whole genome length of *W. sagaensis* ZD-66 was 1,862,304 bp, with a GC content of 36.87%, while GC content was similar to *W. sagaensis* X0750 [29]. The total repetitive sequence length was 5,931 bp. There were 1,848 coding genes, 9 copies of 5S rRNA, 8 copies of 23S rRNA, 8 copies of 16S rRNA, and 76 tRNA molecules.

**Fig. 2:**
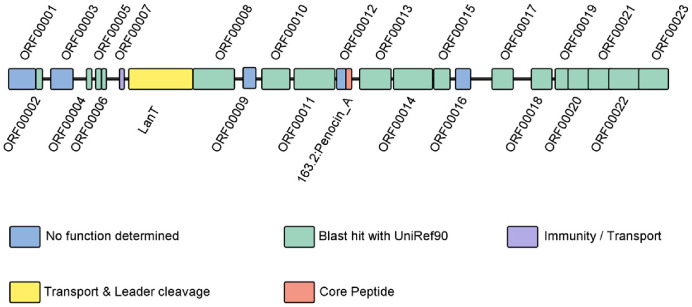
Genetic organization map of bacteriocin gene clusters of *W. sagaensis* ZD-66.

The results of bacteriocin-encoding genes and gene cluster predictions using BAGEL4 are presented in Fig. 2. The cluster included 25 ORFs. The analysis predicted one core peptide Penocin A, a class IIa bacteriocin [30]. Penocin A has a broad antibacterial spectrum and can inhibit various pathogenic bacteria, making it potentially valuable for food safety. Other genes in this cluster include a transport/processing ATP-binding protein (LanT) and a putative bacteriocin Immunity protein (ORF00009).

### Production Kinetics of ZD-66

**Fig. 3:**
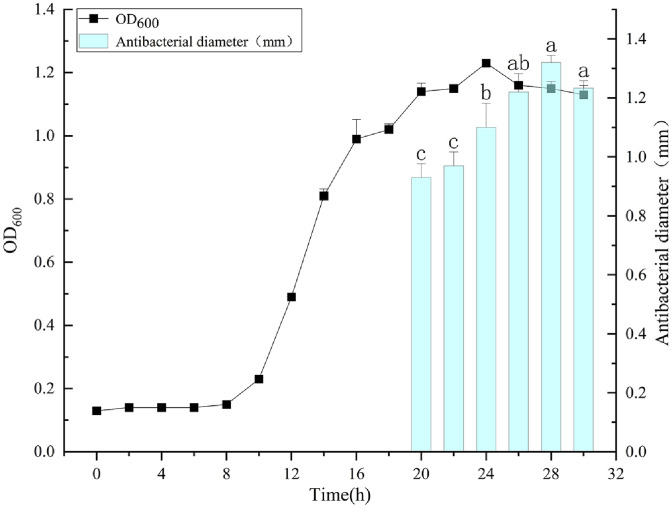
Production of bacteriocin during the growth of *W. sagaensis* ZD-66. Symbols: black triangle, cell number at OD_600_; blue bar, antibacterial activity.

As shown in Fig. 3, the growth curve of ZD-66 showed an “S” shape. The ZD-66 reached the logarithmic phase of growth after 8 h of incubation, with exponential growth from 8-16 h, followed by a stationary phase. The CFS of *W. sagaensis* ZD-66 began to show antimicrobial activity at 20 h, reaching the maximum activity at 28 h and decreased antimicrobial activity after 28 h. This phenomenon may be attributed to the degradation of bacteriocins by endogenous proteases induced by bacteria during growth [21]. Therefore, a cultivation time of 28 h is optimal for bacteriocin production by ZD-66.

### Molecular Mass of ZD-66B

**Fig. 4:**
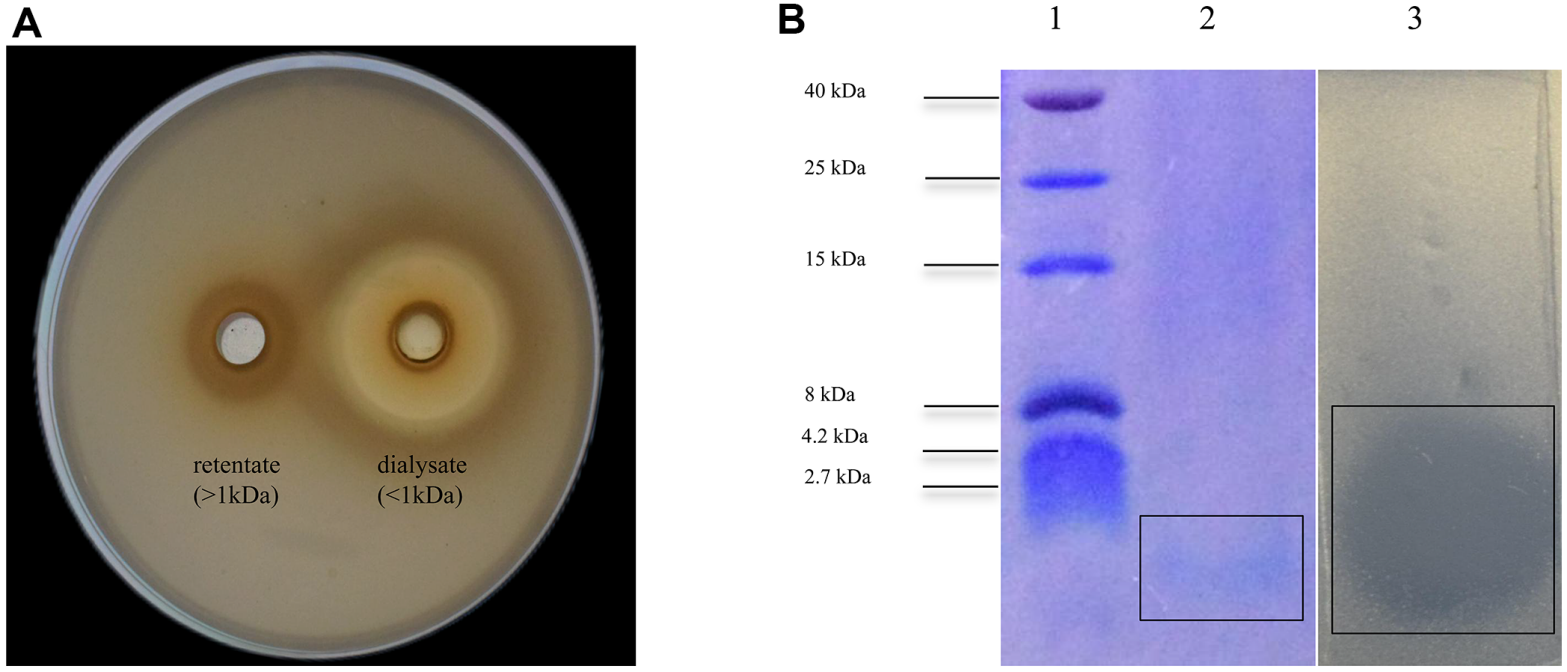
Molecular weight determination of bacteriocin ZD-66B. (**A**) Determination of dialysate and retentate activity of bacteriocin ZD-66B; (**B**) lane 1: Molecular weight marker; lane 2: A single protein band of bacteriocin ZD-66B; lane 3: In situ activity of bacteriocin ZD-66B.

In Fig. 4A, the crude bacteriocin purified by dialysis (component <1 kDa) was shown to have obvious antibacterial effects on the indicator bacteria. Tricine-SDS-PAGE analysis was performed to determine the molecular weight of the ZD-66B. As shown in Fig. 4B, lane 2 formed one sharp band, and there is a clear inhibitory zone at the position of the strip. This result indicates that ZD-66B is an antibacterial substance with a molecular weight of less than 1 kDa.

### Antibacterial Spectrum

As shown in Table 1, the ZD-66B exhibited a broad spectrum of antibacterial activity against Gram-negative and Gram-positive bacteria. Among these pathogens, especially against *S*. Typhimurium ATCC 14028, has the strongest inhibitory activity with a diameter of 24.17 ± 0.30 mm. Overall, bacteriocin ZD-66B demonstrated stronger antibacterial activity against Gram-negative bacteria than against Gram-positive bacteria. However, its antibacterial activity against *L. monocytogenes* ATCC 19115 was stronger than that against *E. coli* ATCC 25922 and *C. sakazakii* SSJ-127. These findings suggest that ZD-66B may have potential applications in food preservation.

### Characteristics of Bacteriocin

**Fig. 5:**
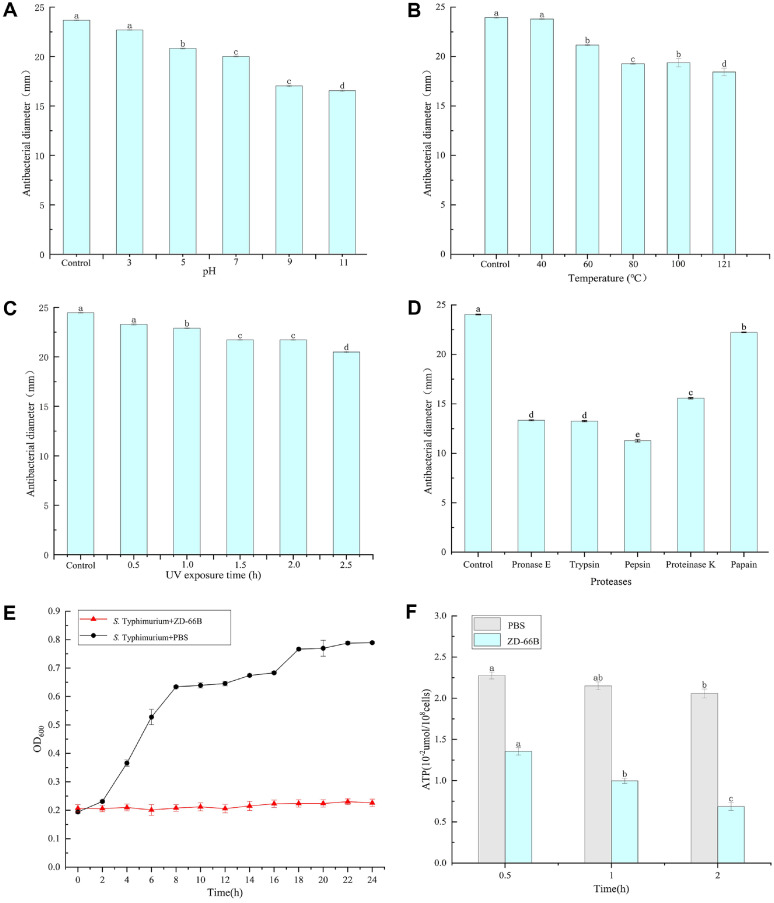
Assessment of the stability and antimicrobial efficacy of bacteriocin ZD-66B. (**A**) Influence of different pH on the antibacterial activity of bacteriocin ZD-66B; (**B**) Influence of temperatures on the antibacterial activity of bacteriocin ZD-66B; (**C**) Influence of UV lights on the antibacterial activity of bacteriocin ZD-66B; (**D**) Influence of different proteases on the antibacterial activity of bacteriocin ZD-66B; (**E**) PBS, and bacteriocin ZD-66B on the growth of *S*. Typhimurium ATCC 14028; (**F**) Intracellular ATP of *S*. Typhimurium ATCC 14028. Different letters in the same table represent significant differences between each parameter (*p* ≤ 0.05).

As shown in Fig. 5, the antibacterial activity of ZD-66B decreased significantly as the pH increased, but the antibacterial activity of ZD-66B at pH 11.0 is still higher than 69.8%. The ZD-66B was sensitive to proteinase K, pronase E, pepsin, and trypsin, while it was resistant to papain. This indicates that ZD-66B has protein properties. The antibacterial activity of ZD-66B after heat treatment is higher than 77.0%. Moreover, ZD-66B demonstrated strong antibacterial activity even at 121°C for 30 min, suggesting its potential for food processing applications. The experimental data demonstrated that continuous ultraviolet radiation exposure progressively decreased the antibacterial efficacy of ZD-66B, with exposure durations of 0.5, 1.0, 1.5, and 2.0 h showing significant reduction (*p* < 0.05). Notably, following 2.5 h of exposure, the ZD-66B retained 83.6% antibacterial activity compared to the untreated control group.

### Time-Kill Curves

In the analysis of the time-kill curves, the growth inhibitory effects of ZD-66B on *S*. Typhimurium ATCC 14028 showed slow inhibition. During the experimental period, the control group demonstrated normal bacterial growth kinetics, whereas ZD-66B treatment exhibited progressively significant growth inhibition against *S*. Typhimurium ATCC 14028 (Fig. 5E). These results indicate that ZD-66B has a time-dependent bactericidal effect on *S*. Typhimurium ATCC 14028.

### Intracellular ATP Concentration

As shown in Fig. 5F, at 0.5 h, the control group intracellular ATP concentration was 0.02276 umol/10^8^ cells. With the raised treatment time, the ATP concentration of *S*. Typhimurium ATCC 14028 significantly decreased in a time-dependence relationship. These results suggest that the bacteriocin ZD-66B may compromise the integrity of the *S*. Typhimurium cell membrane, leading to substantial leakage of ATP.

### Propidium Iodide (PI) Uptake

**Fig. 6:**
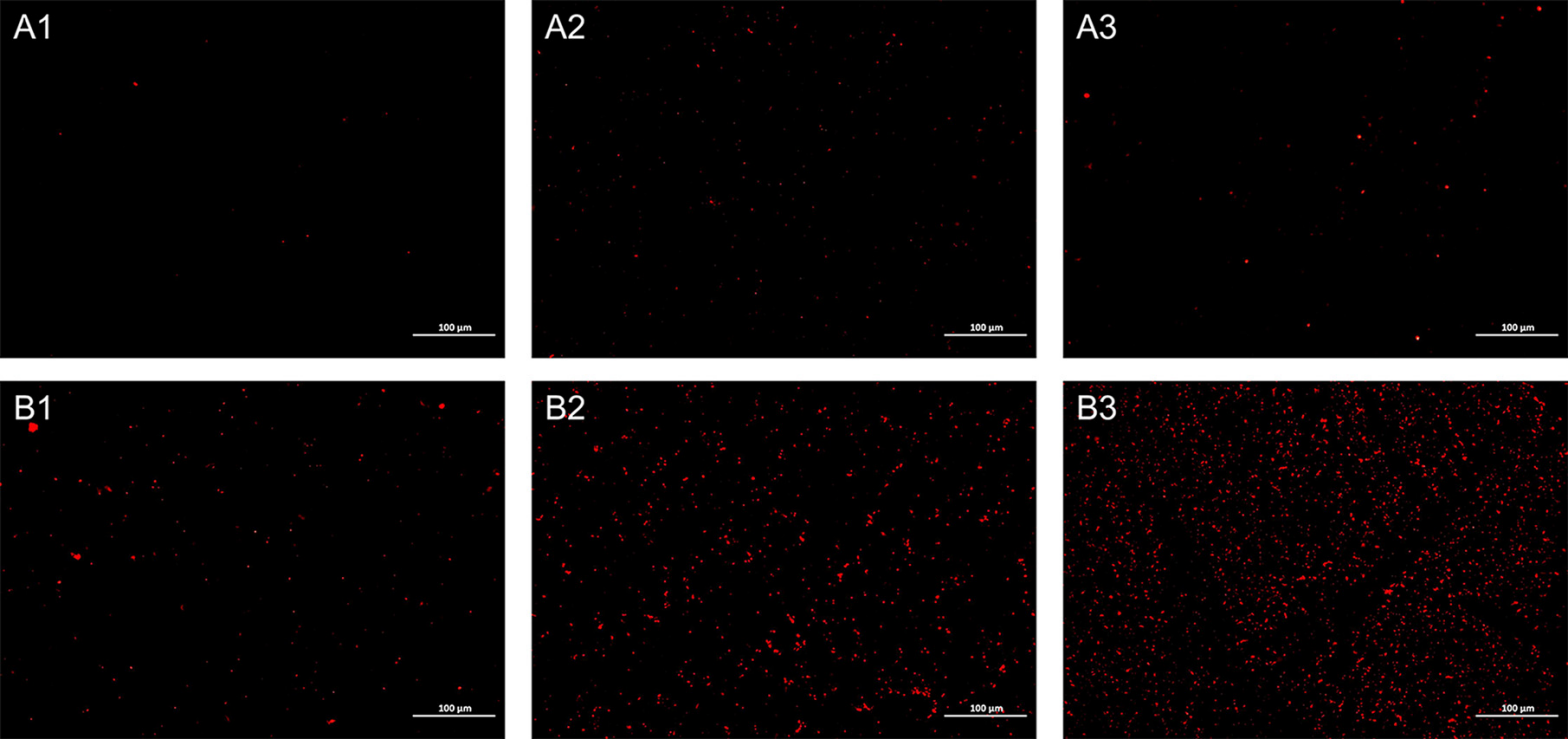
Fluorescence microscope of *S*. Typhimurium ATCC 14028 treated with PBS and bacteriocins. (A1, A2, A3: The PBS was used as a control that treated *S*. Typhimurium ATCC 14028 for 0.5, 1.0 and 2.0 h; B1, B2, B3: The bacteriocin ZD-66B was treated *S*. Typhimurium ATCC 14028 for 0.5, 1.0 and 2.0 h.

PI cannot pass through intact cell membrane. However, upon membrane integrity disruption, PI readily traverses the compromised barrier, binds to DNA, and produces red fluorescence. As shown in Fig. 6, with the increase of time, a small amount of fluorescence appeared in the control group. This is likely due to the natural decay of some bacteria during their growth process. In contrast, treatment with ZD-66B exhibited initial partial red fluorescence after 1.0 h of incubation. Notably, with the fluorescence intensity progressively increasing over time, extensive fluorescence distribution was observed throughout the sample following 2.0 h of incubation. These findings demonstrate that the inhibitory effect of ZD-66B on *S*. Typhimurium ATCC 14028 is time-dependent, and ZD-66B can cause damage to *S*. Typhimurium ATCC 14028 cell membrane.

### Microstructure Analysis by SEM

**Fig. 7:**
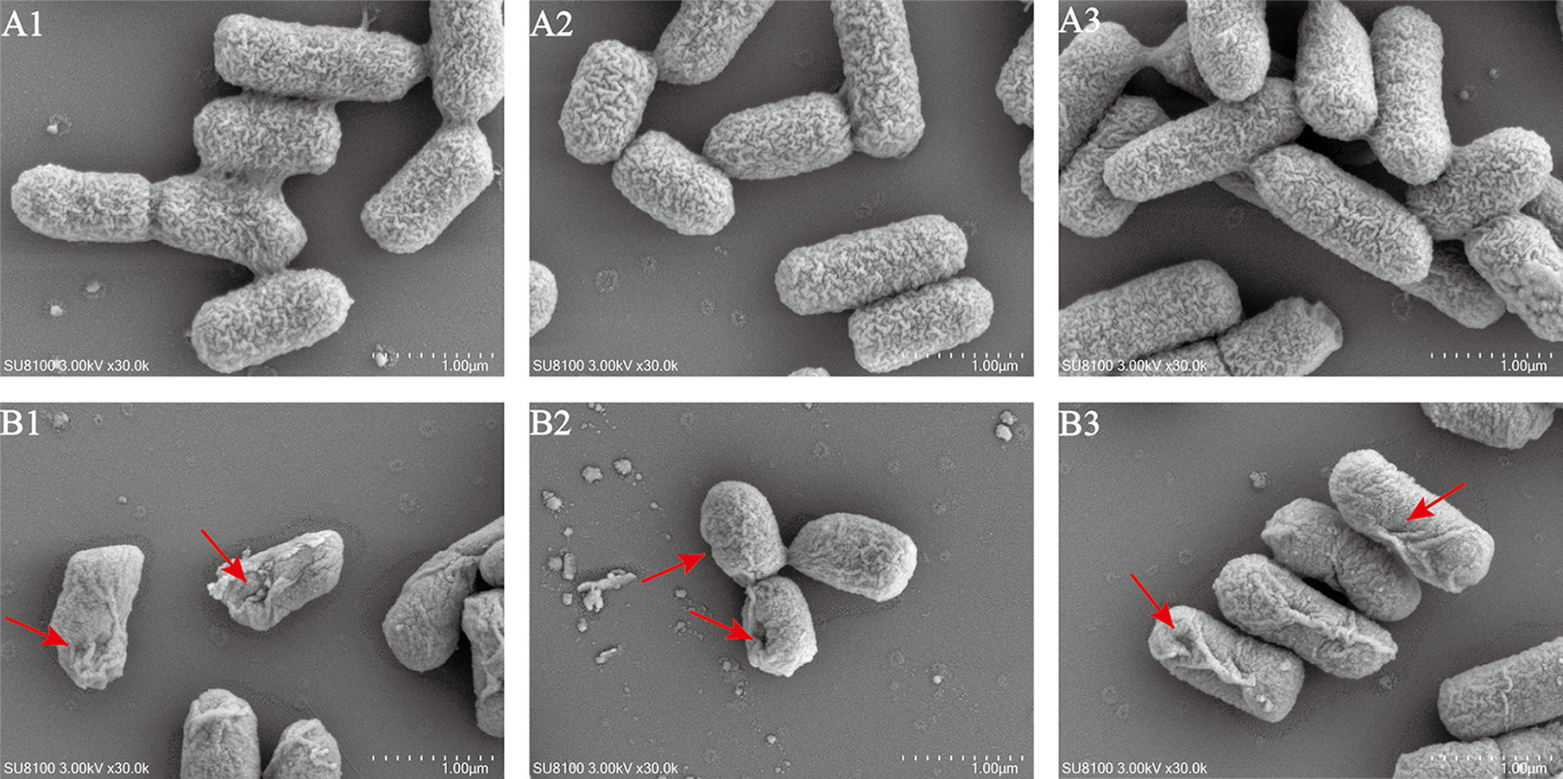
SEM images of *S*. Typhimurium ATCC 14028 before and after bacteriocins treatment. (A1, A2, A3: The PBS was used as a control that treated *S*. Typhimurium ATCC 14028 for 2.0 h; B1, B2, B3: The bacteriocin ZD-66B was treated *S*. Typhimurium ATCC 14028 for 2.0 h. (The results shown in the figure are the shooting results of the same sample at different angles).

To observe the membrane integrity and morphological characteristics by SEM analysis. As shown in Fig. 7, *S*. Typhimurium ATCC 14028 untreated with ZD-66B had intact rod-shaped morphology, smooth cell membrane, and clear outlines. In contrast, after ZD-66B treatment for 2.0 h, the membrane surfaces of *S*. Typhimurium ATCC 14028 showed cell lysis, and the surface became extremely unsmooth, with obvious holes and creping. This suggested that the ZD-66B may be targeted to attack the bacterial membrane and caused cell membrane rupture and the formation of pores.

## Discussion

Salmonella infection is an important public health concern, has enormous economic impact on the health systems [31]. Currently, *Salmonella* antibiotic resistance has led to an increasing risk of treatment failure [32]. Therefore, there is a need for develop new antibacterial agents to replace existing antibiotics. In this study, we demonstrated the bacteriocin antibacterial mechanism of *W. sagaensis* ZD-66 isolated from pickled vegetables. Strain ZD-66 species-level identification was identified using 16S rDNA sequencing. Phylogenetic analysis indicated that strain ZD-66 belonged to *W. sagaensis*. Genome analysis showed the presence of pediocin-like bacteriocin-encoding genes penocin A, a class IIa bacteriocin. Penocin A has a broad antimicrobial spectrum, which is important for food industries [30]. Maximum inhibitory activity was observed after 28 h of incubation at 37°C. The maximum production of bacteriocins occurs during the stable growth phase, indicating that it may be a secondary metabolite [33]. Currently, many bacteriocins produced of *Weissella* spp. with different molecular weights have been reported, such as weissellicin D (62,425.763 Da), *W. hellenica* 4-7(3,205.64 Da) and Weissellin A(4448.9 Da) [24, 33, 34]. In this study, dialysis and SDS-PAGE analysis showed that the bacteriocin produced by *W. sagaensis* ZD-66 has a molecular weight of less than 1 kDa, smaller than those of previously reported bacteriocins produced by Weissella strains. Besides, low-molecular-weight bacteriocin such as bifidocin A (1,198.68 Da), plantaricin JLA-9 (1,044 Da), plantaricin GZ1-27 (975 Da), and *Lactobacillus plantarum* NMGL2 (761.96 Da) are also reported [19, 35, 36, 37]. To our knowledge, the present study is the first discovery of the bacteriocin of *W. sagaensis* ZD-66 has antimicrobial activity against *S*. Typhimurium ATCC 14028. A similar result was reported for *Weissella paramesenteroids* J1 isolated from the poultry intestine [4]. Furthermore, the bacteriocin of *W. sagaensis* ZD-66 found has a broad antibacterial spectrum, inhibiting *E. coli*, *S. aureus*, *C. sakazakii* and *L. monocytogenes*. Similar results were also reported for the bacteriocin *Lactobacillus crustorum* MN047 [26]. Therefore, the broad antibacterial spectrum of *W. sagaensis* ZD-66 means it’s a good candidate for food preservation.

The results of temperature tests showed that bacteriocin ZD-66B retained approximately 76% of its original antibacterial activity after treatment at 121°C for 30 min. Similar results have been reported for other bacteriocins [38, 39]. This thermal stability may be related to the hydrophobic tertiary structure of the peptides [19]. Conversely, the bacteriocin CV7 has been reported to be inactivated at temperature of 121°C for 30 min [40]. Some studies have revealed that the activity of pentocin MQ1, *L. plantarum* M.2 and *Lactobacillus curvatus* B.67 showed no residual antimicrobial activity after treatment at pH 10 [41, 42]. In contrast, the antibacterial activity of bacteriocin ZD-66B was more stable at pH 11. The bacteriocin ZD-66B showed good stability under UV light, similar to *L. plantarum* NMGL2 [19]. The bacteriocin from *W. sagaensis* ZD-66 was sensitive to pepsin, causing 53% loss of activity, compared with the untreated control group. However, there was little reduction after treated with papain, which is similar to the results reported by [43]. This is probably because the papain preferentially cleaves the site of containing the catalytic triad (Ser-His-Asp) [44]. The bacteriocin ZD-66B showed great utilization potential as a food preservation, because of the thermal and pH stabilities of it.

To further explore the mode of action of bacteriocin ZD-66B, through bactericidal kinetics curves, propidium iodide (PI) uptake, intracellular ATP content, and scanning electron microscopy (SEM). Time-kill curve experiments showed that bacteriocin ZD-66B expressed inhibition against *S*. Typhimurium ATCC 14028. This result was similar to bacteriocin LpH25 [45]. PI uptake showed began to exhibit red fluorescence after incubation for 1.0 h, extensive fluorescence distribution after 2.0 h of incubation, with the fluorescence intensity progressively increasing over time. Thus, the bacteriocin ZD-66B damaged the integrity of cell membrane. Similar results were also reported by Zhang *et al*. [46]. ATP is the source of energy for bacterial growth and reproduction [47]. With increasing bacteriocin ZD-66B treatment time, intracellular ATP levels significantly reduced. This result is consistent with other studies [27]. It is possible that bacteriocin ZD-66B changes the cell membrane, resulting in leakage of ATP, ultimately triggering apoptosis. *S. enterica* ATCC 14028 cells treated with the ZJ316 from *L. plantarum* showed destroyed structures and dissolved cells [48]. Under SEM, we observed that treatment with bacteriocin ZD-66B resulted in cell lysis, rough surfaces, and pore formation. Antibacterial modes of bacteriocin include inhibition of the cell wall, protein synthesis, and pore formation, etc. [43]. The pore formation can cause severe membrane permeability and ion leakage (protein and nucleic acid) [39]. All in all, these results confirmed that the mode of action of bacteriocin ZD-66B is pore formation and finally cell death.

## Conclusion

This study clearly demonstrated that bacteriocin ZD-66B can inhibit the growth of *S*. Typhimurium ATCC 14028, and exhibits great physicochemical characteristics including thermal stability, acid-base tolerance, and UV stability. Furthermore, bacteriocin ZD-66B is a class II bacteriocin with a molecular weight of less than 1 kDa. Bacteriocin ZD-66B was found to disrupt and form pores cell membrane of *S*. Typhimurium ATCC 14028, thereby eliminating the bacteria. These findings suggest the bacteriocin ZD-66B has the potential as a promising bio-preservative for food preservation. However, the purification and structural analysis of bacteriocin ZD-66B still needs to be further studied.

## Figures and Tables

**Table 1:** The antimicrobial spectrum of ZD-66B.

Indicator strain	Source	Gram	Inhibition zone (mm)
*S.* Typhimurium	ATCC 14028	G^-^	24.17 ± 0.30^a^
*E. coli*	ATCC 25922	G^-^	21.53 ± 0.44^c^
*S. aureus*	ATCC 25923	G^+^	20.54 ± 0.23^d^
*C. sakazakii*	SSJ-127	G^-^	22.62 ± 0.97^b^
*L. monocytogenes*	ATCC 19115	G^+^	22.18 ± 0.16^bc^

The diameter of the antibacterial zone includes the diameter of the Oxford cup. The significant difference (*p* ≤ 0.05) was indicated by different letters between each parameter.
